# Optimising Therapeutic Intervals for Anti-TNF Biologics in Rheumatoid Arthritis: A Retrospective Real-World Study

**DOI:** 10.3390/life16050723

**Published:** 2026-04-24

**Authors:** Jose Manuel Dodero-Anillo, Manuel Rosety-Rodriguez, Maria Jose Pedrosa-Martinez

**Affiliations:** 1 Clinical Pharmacology, Hospital Universitario Puerto Real, 11510 Cadiz, Spain; 2 Faculty of Medicine, University of Cadiz, 11009 Cadiz, Spain

**Keywords:** rheumatoid arthritis, biologics, therapeutic drug monitoring, infliximab, adalimumab, etanercept, anti-drug antibodies, DAS28, therapeutic interval

## Abstract

Background: Therapeutic drug monitoring (TDM) of anti-TNF biologics may help optimise treatment in rheumatoid arthritis (RA), but current therapeutic ranges do not always reflect clinical response in routine practice. Methods: We conducted a retrospective observational study including 224 adults with RA treated with infliximab (IFX), adalimumab (ADL), or etanercept (ETN) during the maintenance phase at Hospital Universitario Puerto Real between May 2016 and May 2023. Drug and anti-drug antibody levels were measured by sandwich ELISA and analysed against clinical response using DAS28. Demographic and clinical variables, associations between drug levels and response, the effect of antibodies, correlations between serum concentrations and DAS28, and the performance of current and proposed therapeutic ranges were evaluated. Results: The cohort was mainly female (62.1%), with a mean age of 57.7 years, BMI of 28.5 kg/m^2^, and mean DAS28 of 3.33. ETN was most frequently used (62.5%), followed by ADL (21.9%) and IFX (15.6%). Drug levels were significantly associated with response (*p* < 0.001). Anti-drug antibodies were strongly linked to non-response, especially with IFX and ADL. Serum drug levels correlated inversely with disease activity for IFX and ADL, but not for ETN. Current therapeutic ranges showed low sensitivity, while lower proposed ranges improved sensitivity considerably. Conclusions: Current anti-TNF therapeutic ranges have limited ability to identify responders in real-world RA. Lowering the lower bound improves sensitivity and supports more individualised TDM, particularly for IFX and ADL, pending prospective validation.

## 1. Introduction

Rheumatoid arthritis (RA) is a chronic inflammatory autoimmune disease characterised by persistent synovitis, progressive joint damage, and systemic complications that significantly impact quality of life and long-term outcomes [1,2,3]. Although epidemiological estimates vary depending on the population studied, RA affects approximately 0.5–1% of adults worldwide, representing a substantial burden for healthcare systems [2,3]. The pathogenesis of RA involves a complex interplay between genetic susceptibility, environmental factors, and immune dysregulation, leading to sustained inflammation and structural joint damage [4,5,6,7]. Among the key mediators, tumour necrosis factor alpha (TNF-α) plays a central role by amplifying inflammatory pathways and promoting tissue destruction [8,9].

The introduction of biologic disease-modifying antirheumatic drugs (bDMARDs), particularly TNF inhibitors such as infliximab (IFX), adalimumab (ADL), and etanercept (ETN), has significantly improved disease control and functional outcomes in patients with RA [10,11,12]. Despite their effectiveness, a considerable proportion of patients do not achieve sustained remission or low disease activity, and some experience a loss of response over time. This variability in therapeutic outcomes has been attributed, in part, to inter-individual differences in pharmacokinetics and to the development of anti-drug antibodies, which may increase drug clearance or neutralise its biological activity [13,14,15,16]. Additionally, patient-related factors such as smoking, obesity, and prior biologic exposure may further influence treatment response [17,18,19,20,21,22,23].

These challenges have led to increasing interest in therapeutic drug monitoring (TDM) as a strategy to optimise biologic therapy in RA. By measuring serum drug concentrations and anti-drug antibodies, TDM aims to support clinical decision-making, guide dose adjustments, and improve treatment efficiency [13,14,15,16,24]. However, the clinical implementation of TDM in RA remains a matter of debate. One of the main limitations is that currently accepted therapeutic ranges are often derived from heterogeneous populations or controlled clinical trial settings with strict inclusion and exclusion criteria, fixed dosing regimens, and limited variability in concomitant treatments, which may not fully reflect routine clinical practice [14,24,25,26,27]. As a consequence, discrepancies between drug levels and clinical response are frequently observed, with some patients achieving adequate disease control despite having concentrations below the accepted range, while others remain active despite levels within it.

This mismatch raises an important clinical question: are the currently used therapeutic intervals adequately calibrated for real-world populations, or do they require adjustment to better reflect actual treatment response? Addressing this issue is essential to improve the clinical utility of TDM and to advance towards a more individualised approach to RA management.

In this context, the present study was conducted in a real-world cohort of patients with RA receiving anti-TNF therapy in routine clinical practice. Using serum drug and anti-drug antibody measurements together with clinical response assessed by DAS28, we aimed to evaluate the relationship between pharmacokinetic variables and treatment outcomes. Furthermore, we assessed the performance of currently used therapeutic intervals and explored alternative ranges more closely aligned with observed clinical response. We hypothesised that recalibrating therapeutic intervals based on real-world data would improve their discriminatory capacity and provide a more useful framework for treatment optimization.

## 2. Materials and Methods

This retrospective observational study was conducted at Hospital Universitario Puerto Real, a tertiary public hospital that provides care to a reference population of 326,674 inhabitants in the Bahia de Cadiz-La Janda district [28]. In the absence of a dedicated rheumatology department, patients with RA receiving biologic therapy in our catchment area are managed by the Internal Medicine service. All TDM analyses were performed in the Clinical Pharmacology unit as part of the hospital multidisciplinary biologics committee.

We reviewed all anti-TNF TDM determinations performed between May 2016 and May 2023 and selected adult patients with a diagnosis of RA who were receiving IFX, ADL, or ETN in the maintenance phase, defined as at least 6 months of uninterrupted treatment. Patients younger than 18 years, those in induction, and those with insufficient clinical information to classify therapeutic response were excluded. To avoid analytical heterogeneity, patients treated with biosimilars were not included. When a patient changed from one anti-TNF agent to another, each maintenance treatment episode was analysed within the corresponding drug-specific group, although the patient was no longer considered biologic-naive.

Drug and anti-drug antibody concentrations were measured using a sandwich ELISA assay in the Clinical Pharmacology laboratory [29]. Clinical and demographic information was extracted retrospectively from the Andalusian Health System electronic records (DIRAYA) and from the TDM records maintained by the Clinical Pharmacology unit. Data were pseudonymised before analysis in accordance with institutional and regional data protection requirements.

The primary outcome was treatment response, defined clinically according to maintenance of favourable disease evolution without need for dose escalation or change of biologic, together with DAS28 category. In practical terms, patients with remission or low disease activity, or with clinically documented improvement reflected by a reduction in DAS28 category or symptoms after anti-TNF therapy, were classified as responders, whereas patients with stable or worsening disease activity requiring treatment modification were considered non-responders. DAS28 values were interpreted as remission (<2.6), low activity (2.6 to <3.2), moderate activity (3.2–5.1), and high activity (>5.1) [12,30].

Collected quantitative variables were age, BMI, DAS28, serum drug level, and serum anti-drug antibody level. Qualitative variables included sex, smoking status, family history of RA, drug prescribed, prior biologic exposure (naive vs. non-naive), concomitant methotrexate use, categorical drug level (subtherapeutic, therapeutic, supratherapeutic), categorical anti-drug antibody result (negative vs. positive), and therapeutic response. Anti-drug antibody negativity was defined by the laboratory thresholds used in routine care: <2 U/mL for IFX, <3.12 U/mL for ADL, and <5 U/mL for ETN.

Sample size was estimated using G*Power version 3.1.9.7 with a statistical power of 95%, alpha error of 0.05, and 10% anticipated losses, yielding a minimum sample of 213 subjects. The final study population included 224 patients, thereby exceeding the planned sample size.

Descriptive statistics were calculated as mean and standard deviation for continuous variables and as absolute frequencies and percentages for categorical variables. Normality was assessed with Kolmogorov–Smirnov and Shapiro–Wilk tests. Because BMI, drug levels, anti-drug antibody levels, and DAS28 showed non-parametric distributions, non-parametric methods were used where appropriate. Associations between categorical variables were analysed using Pearson chi-square or likelihood ratio tests. Group comparisons involving non-parametric continuous variables were performed with the Mann–Whitney U test. Correlations between serum drug levels and DAS28 were examined using Spearman’s rho.

For the diagnostic performance analysis, established therapeutic intervals were compared with new intervals derived from the observed distribution of responders and non-responders. The established intervals used in our setting were 3–8 μg/mL for IFX, 5–8 μg/mL for ADL, and 2–3 μg/mL for ETN [24,25,26,27]. Proposed new intervals were 1–8 μg/mL for IFX, 2–10 μg/mL for ADL, and 1–5 μg/mL for ETN. For each drug, we calculated sensitivity, specificity, positive predictive value (PPV), and negative predictive value (NPV), considering responders within range as true positives and non-responders out of range as true negatives. Statistical analyses were performed with IBM SPSS Statistics 23. A two-sided *p*-value < 0.05 was considered statistically significant.

## 3. Results

A total of 224 patients with rheumatoid arthritis were included in the analysis. The mean age was 57.71 ± 9.75 years (range 36–87), with a predominance of women (62.1%) over men (37.9%). The mean body mass index (BMI) was 28.50 ± 3.89 kg/m^2^, placing the cohort on average in the overweight range, and the mean DAS28 score was 3.33 ± 1.24, consistent with overall moderate disease activity. Smoking was recorded in 37.1% of patients, and 30.4% reported a family history of rheumatoid arthritis. Most treatment episodes corresponded to biologic-naive patients (69.6%), whereas 30.4% had previously received another biologic agent.

Regarding treatment distribution, etanercept (ETN) was the most frequently prescribed anti-TNF agent (140 patients; 62.5%), followed by adalimumab (ADL, 49 patients; 21.9%) and infliximab (IFX, 35 patients; 15.6%). Notably, 95% of ETN-treated patients were biologic-naive, suggesting that ETN was preferentially used as first-line biologic therapy in routine clinical practice during the study period. Baseline demographic and clinical characteristics are summarised in Table 1.

Normality testing showed that age followed a normal distribution, whereas BMI, DAS28, serum drug levels, and anti-drug antibody levels did not. There were no significant differences in age distribution between men and women (*p* = 0.840), indicating that both groups were comparable in terms of age.

In the overall cohort, categorical serum drug levels were significantly associated with treatment response (chi-square = 20.301, *p* < 0.001). Subtherapeutic concentrations were more frequent among non-responders (71.6%) compared with responders (38.9%). Conversely, therapeutic and supratherapeutic levels were more commonly observed among responders, supporting a clear relationship between drug exposure and clinical outcome. When analysed by individual drug, this association remained statistically significant for IFX (likelihood ratio *p* = 0.012) and ADL (likelihood ratio *p* = 0.001), but not for ETN (chi-square *p* = 0.078).

In the IFX group (n = 35), 24 patients were classified as responders and 11 as non-responders, corresponding to an overall response rate of 68.57%. Within this group, 48.6% of patients had subtherapeutic drug levels, 37.1% had therapeutic levels, and 14.3% had supratherapeutic levels. Among non-responders, subtherapeutic concentrations were particularly frequent (81.8%). In contrast, responders were more evenly distributed, with a higher proportion showing therapeutic or supratherapeutic levels. Anti-drug antibody status showed a strong association with response: 72.7% of non-responders were antibody-positive, whereas 95.8% of responders were antibody-negative (*p* < 0.001).

In the ADL group (n = 49), 31 patients were responders and 18 were non-responders, yielding a response rate of 63.26%. Subtherapeutic concentrations were observed in 59.2% of patients overall and were especially frequent among non-responders (88.9%). Among responders, the distribution was more balanced, with 41.9% subtherapeutic, 29.0% therapeutic, and 29.0% supratherapeutic levels. Anti-drug antibodies were detected in 44.4% of non-responders and were not observed in responders, again demonstrating a strong association between immunogenicity and treatment failure (*p* < 0.001).

The ETN group included 140 patients, of whom 102 were responders and 38 non-responders, corresponding to the highest response rate among the three drugs (72.85%). The distribution of ETN levels differed from that of IFX and ADL: 45.0% of patients had subtherapeutic levels, 22.9% therapeutic levels, and 32.1% supratherapeutic levels. Although responders tended to have higher concentrations, the association between categorical ETN levels and response did not reach statistical significance. Furthermore, no anti-ETN antibodies were detected in this group.

In the pooled analysis, anti-drug antibodies were present in 7.6% of patients and were strongly associated with treatment failure (chi-square = 36.175, *p* < 0.001). This association remained significant in both the IFX and ADL groups, whereas it could not be evaluated in ETN due to the absence of detectable antibodies.

We also explored the potential influence of concomitant methotrexate on pharmacokinetic variables. In the IFX group, methotrexate use was not significantly associated with serum drug concentrations (*p* = 0.959) or antibody levels (*p* = 0.130). In contrast, in the ADL group, methotrexate use was associated with higher serum drug levels (*p* = 0.019), although the association with antibody formation did not reach statistical significance (*p* = 0.175). No significant associations were observed in the ETN group.

Spearman correlation analysis showed an inverse relationship between serum drug levels and disease activity as measured by DAS28. In the overall cohort, the correlation coefficient was −0.421 (*p* < 0.001), indicating that higher drug concentrations were generally associated with lower disease activity. This relationship was stronger for IFX (rho = −0.615; *p* < 0.001) and ADL (rho = −0.680; *p* < 0.001), supporting a clinically relevant exposure–response relationship. In contrast, the correlation observed for ETN was weaker (rho = −0.261) and of limited clinical relevance.

Clinical variables were also examined. Smoking was significantly associated with poorer response, with 53.7% of non-responders being smokers compared with 29.9% of responders (*p* = 0.001). Higher BMI was also associated with reduced treatment response (*p* = 0.005). In contrast, family history of RA was not significantly associated with therapeutic outcomes (*p* = 0.458).

Finally, the diagnostic performance of currently used therapeutic intervals was evaluated and compared with proposed alternative ranges. For IFX, the established interval (3–8 µg/mL) showed a sensitivity of 0.46, specificity of 0.82, positive predictive value (PPV) of 0.85, and negative predictive value (NPV) of 0.41. Lowering the lower bound to 1 µg/mL increased sensitivity to 0.61 (*p* = 0.024), with a moderate reduction in specificity (0.75). For ADL, the standard interval (5–8 µg/mL) yielded a sensitivity of 0.29 and specificity of 0.89, whereas the proposed interval (2–10 µg/mL) improved sensitivity to 0.61 (*p* = 0.004) with acceptable predictive values. The ETN interval (2–3 µg/mL) showed particularly low sensitivity (0.25), which increased to 0.75 when expanded to 1–5 µg/mL (*p* = 0.047), although specificity decreased to 0.50. These results are summarised in Table 2 and Table 3 and indicate that lowering the lower bound of therapeutic intervals improves their ability to identify responders in real-world clinical practice.

## 4. Discussion

This study provides a detailed real-world assessment of anti-TNF TDM in RA and suggests that currently applied therapeutic intervals may be insufficiently calibrated for routine clinical use in our population. The main finding is that the lower limit of accepted serum concentrations appears overly restrictive for all three drugs, leading to a substantial number of clinically responding patients being classified as subtherapeutic. By redefining therapeutic intervals as 1–8 μg/mL for IFX, 2–10 μg/mL for ADL, and 1–5 μg/mL for ETN, we observed clear gains in sensitivity, while preserving a clinically acceptable predictive profile.

The descriptive profile of our cohort is consistent with the known epidemiology of RA. The predominance of women and the age distribution are in line with national data showing that RA is more frequent in women and typically diagnosed in midlife [2,3,31]. Although age and sex were recorded as baseline variables, the present study was not specifically designed to evaluate their influence on treatment response. Therefore, stratified analyses were not performed, which may represent a limitation. Future studies with larger sample sizes and dedicated designs should further explore the potential impact of demographic factors on anti-TNF response. The high prevalence of smoking and overweight is also clinically relevant because both factors have been implicated not only in disease susceptibility but also in persistence of inflammation and poorer therapeutic outcomes [17,18,19,20,21,22,23,32,33]. In our study, smoking was clearly associated with non-response, reinforcing previous evidence that active smokers respond less well to methotrexate and TNF inhibitors and may require more intensive treatment [19,20,21,22,23]. Likewise, overweight was associated with poorer response, which supports the increasingly recognised role of adiposity-driven inflammation in RA [17].

The biologic prescribing pattern observed in our cohort is also informative. ETN was the predominant first-line anti-TNF agent, whereas IFX and ADL were more commonly used after failure of a previous biologic. This therapeutic sequence likely reflects local prescribing habits as well as the perception of ETN as a safer or more practical first biologic in our setting. Such differences are important when interpreting exposure–response relationships because a cohort enriched with biologic-experienced patients may include more refractory disease and a higher baseline risk of treatment failure.

The strongest exposure–response relationships in our study were found for IFX and ADL. Both drugs showed significant associations between categorical concentration and therapeutic response, as well as moderately strong inverse correlations with DAS28. These findings are coherent with previous reports showing that lower serum IFX or ADL concentrations and anti-drug antibody development are associated with loss of response [25,26,27]. The observation that antibody-positive patients were overwhelmingly non-responders further supports the biological plausibility of TDM for monoclonal anti-TNF agents. In clinical terms, IFX and ADL TDM seems particularly useful when confronted with secondary non-response, as it may help distinguish underexposure or immunogenicity from pharmacodynamic failure.

ETN behaved differently. No patient developed detectable anti-ETN antibodies, and serum ETN levels did not correlate clearly with DAS28. This pattern is compatible with the lower immunogenicity traditionally attributed to ETN and with prior studies showing a weaker or less consistent concentration–response relationship for this fusion protein than for monoclonal antibodies [34]. Even so, our results are clinically important because they indicate that the currently accepted ETN interval may exclude many responders. The striking improvement in sensitivity after expanding the ETN interval to 1–5 μg/mL suggests that, in practice, many clinically stable patients can remain well controlled at serum levels lower than previously assumed.

The diagnostic performance analysis is arguably the most relevant contribution of the present work. Current intervals yielded sensitivities of 46% for IFX, 29% for ADL, and 25% for ETN, indicating that they identify only a minority of true responders as being in range. In other words, reliance on these thresholds may drive unnecessary dose escalation or inappropriate switching in patients who are actually responding. The proposed intervals substantially improved sensitivity to 61%, 61%, and 75%, respectively. Although this came at the cost of some reduction in specificity, particularly for ETN, the trade-off appears clinically reasonable in a tool intended primarily to support, rather than replace, clinical judgement.

The effect of concomitant immunomodulators, particularly methotrexate, on reducing immunogenicity is well established. Methotrexate is thought to suppress B-cell activation and proliferation, thereby limiting the formation of anti-drug antibodies. This reduction in immunogenicity decreases immune-mediated drug clearance and results in higher and more stable circulating biologic concentrations. In addition, methotrexate may modulate T-cell responses and cytokine production, further contributing to a more favourable pharmacokinetic and pharmacodynamic profile of anti-TNF agents. These mechanisms help explain the improved drug exposure and clinical response observed in patients receiving combination therapy. In our work, we observed a significant association between concomitant methotrexate and higher ADL levels, which is consistent with literature suggesting that immunomodulators may reduce immunogenicity and improve biologic exposure [16,26]. However, corresponding associations were not demonstrated for IFX or ETN in our dataset. This lack of significance may reflect limited sample size in the IFX group, different prescribing patterns, or true drug-specific effects. It also illustrates why local validation matters: assumptions derived from other diseases or populations do not necessarily translate directly to every RA cohort.

Several limitations must be acknowledged. First, the retrospective and single-centre design limits causal inference and may reduce generalisability. Second, treatment episodes rather than unique patients were analysed in drug-specific groups when therapy changed over time, which better reflects clinical reality but may complicate strict independence assumptions. Third, the study focused on effectiveness and exposure–response relationships, but did not capture adverse events in a systematic way, so the upper bound of the proposed intervals should be interpreted cautiously. Fourth, because ETN antibody positivity was absent in our sample, immunogenicity conclusions for ETN remain limited to the observation of apparent non-immunogenic behaviour in this cohort. Another limitation of this study is the lack of longitudinal follow-up to evaluate the duration of therapeutic response. Given the retrospective and cross-sectional nature of the data, response was assessed at the time of therapeutic drug monitoring rather than over time. Future prospective studies incorporating time-to-event analyses would provide a more comprehensive understanding of treatment durability. Finally, the study was conducted in a setting without biosimilar exposure, and our results should therefore not be extrapolated directly to mixed originator–biosimilar populations.

Despite these limitations, the study has important strengths. It includes a relatively large real-world cohort with a pre-specified sample size, uses a consistent analytical platform for all TDM determinations, evaluates clinically meaningful response rather than laboratory surrogates alone, and integrates drug-specific, antibody-related, and patient-related factors. Most importantly, it provides a pragmatic framework for how local TDM data can be translated into more useful therapeutic intervals.

From a clinical perspective, our data support three practical messages. First, TDM is most informative for IFX and ADL, where serum levels and immunogenicity show clear relationships with disease activity and response. Second, for ETN, TDM may still be useful, but interpretation should be more cautious and less dependent on narrow historical intervals. Third, TDM results should never be interpreted in isolation: smoking status, BMI, treatment history, and methotrexate co-therapy all shape the clinical context in which concentration measurements are meaningful. Prospective multicentre studies with repeated measurements and formal safety assessment are now needed to determine whether adopting these revised intervals improves patient outcomes, reduces unnecessary dose escalation, and optimises biologic resource utilisation.

## 5. Conclusions

In this retrospective real-world study of 224 maintenance-phase anti-TNF treatment episodes in RA, current therapeutic intervals for IFX, ADL, and ETN show limited sensitivity for identifying true responders.

For IFX and ADL, serum drug levels were inversely correlated with DAS28 and anti-drug antibodies were strongly associated with treatment failure, supporting the clinical utility of TDM for both monoclonal antibodies. ETN showed a different profile, with no detectable anti-drug antibodies and no clear concentration-DAS28 correlation, but still benefitted from interval recalibration.

Therapeutic intervals of 1–8 μg/mL for IFX, 2–10 μg/mL for ADL, and 1–5 μg/mL for ETN improved the sensitivity of TDM and better matched clinical outcomes in our cohort. Smoking and overweight were also associated with poorer response and should be addressed as part of comprehensive management.

These findings support a more individualised interpretation of anti-TNF TDM in RA and provide a rationale for prospective validation studies aimed at defining clinically robust, population-adapted therapeutic intervals.

## Figures and Tables

**Table 1:** Demographic and baseline clinical characteristics of the cohort. (Abbreviations: BMI, body mass index; DAS28, Disease Activity Score 28; RA, rheumatoid arthritis.).

Variable	Value
Age (years)	57.71 ± 9.75 (36–87)
BMI (Kg/m^2^)	28.50 ± 3.89
DAS28	3.33 ± 1.24 (0.49–6.21)
Women	139/224 (62.1%)
Smokers	83/224 (37.1%)
Family history of RA	68/224 (30.4%)
Biologic-naive	156/224 (69.6%)

**Table 2:** Response rates and categorical serum drug concentrations according to anti-TNF agent. (Abbreviations: IFX, infliximab; ADL, adalimumab; ETN, etanercept. Subtherapeutic, therapeutic, and supratherapeutic levels defined according to established therapeutic ranges).

Drug	Total (n)	Responders n (%)	Non-Responders n (%)	Subtherapeutic (%)	Therapeutic (%)	Supratherapeutic (%)
IFX	35	24 (68.57)	11 (31.43)	48.6	37.1	14.3
ADL	49	31 (63.26)	18 (36.74)	59.2	22.4	18.4
ETN	140	102 (72.85)	38 (27.15)	45.0	22.9	32.1

**Table 3:** Diagnostic performance of established and proposed therapeutic intervals. (Abbreviations: IFX, infliximab; ADL, adalimumab; ETN, etanercept. PPV, positive predictive value; NPV, negative predictive value).

Drug	Interval	Sensitivity	Specificity	PPV	NPV
IFX	3–8 µg/mL	0.46	0.82	0.85	0.41
ADL	5–8 µg/mL	0.29	0.89	0.82	0.42
ETN	2–3 µg/mL	0.25	0.84	0.81	0.30
IFX (proposed)	1–8 µg/mL	0.61	0.75	0.82	0.50
ADL (proposed)	2–10 µg/mL	0.61	0.72	0.79	0.52
ETN (proposed)	1–5 µg/mL	0.75	0.50	0.80	0.42

## Data Availability

No new data were created or analyzed in this study. Data sharing is not applicable to this article.
